# Statistical methods to correct for verification bias in diagnostic studies are inadequate when there are few false negatives: a simulation study

**DOI:** 10.1186/1471-2288-8-75

**Published:** 2008-11-11

**Authors:** Angel M Cronin, Andrew J Vickers

**Affiliations:** 1 Department of Epidemiology and Biostatistics and Department of Surgery, Memorial Sloan-Kettering Cancer Center, 1275 York Avenue, NY, NY 10021, USA

## Abstract

**Background:**

A common feature of diagnostic research is that results for a diagnostic gold standard are available primarily for patients who are positive for the test under investigation. Data from such studies are subject to what has been termed "verification bias". We evaluated statistical methods for verification bias correction when there are few false negatives.

**Methods:**

A simulation study was conducted of a screening study subject to verification bias. We compared estimates of the area-under-the-curve (AUC) corrected for verification bias varying both the rate and mechanism of verification.

**Results:**

In a single simulated data set, varying false negatives from 0 to 4 led to verification bias corrected AUCs ranging from 0.550 to 0.852. Excess variation associated with low numbers of false negatives was confirmed in simulation studies and by analyses of published studies that incorporated verification bias correction. The 2.5^th ^– 97.5^th ^centile range constituted as much as 60% of the possible range of AUCs for some simulations.

**Conclusion:**

Screening programs are designed such that there are few false negatives. Standard statistical methods for verification bias correction are inadequate in this circumstance.

## Background

A common feature of diagnostic research is that results for a diagnostic gold standard are available only for patients who are positive for the test under investigation. Prostate-specific antigen (PSA) testing is a typical example: we want to know the operating characteristics of the PSA test, but men are only recommended for biopsy (the gold standard assessment of prostate cancer) if their PSA is above a specified threshold, such as 4 ng/ml. Accordingly we have little information on whether men with PSA less than 4 mg/ml do or do not have prostate cancer. True disease state is therefore known for only a subset of participants, and because that subset is determined by the diagnostic test result, data are subject to what has been termed "verification bias"[1]. Verification bias is associated particularly with screening tests: screening a healthy population for a symptomless disease will, by definition, result in further diagnostic work up only for those with a positive screening test.

Begg and Greenes have proposed straightforward Bayesian methods to correct for verification bias [1,2]. Their method has been widely used [3-9] and requires only simple computations to estimate sensitivity and specificity for each threshold of the diagnostic test result, which can then be used to derive the area-under-the-receiver-operating-characteristics-curve (AUC). Alonzo and Pepe recently described another method for computing the AUC of a continuous screening test in the presence of verification bias. This method involves computing the sensitivity and specificity for each observed value of the screening test, but again is straightforward to implement[10]. Both of these methods rely on the assumption that data are missing at random[11], in other words, no other factor besides the diagnostic test result influenced verification status. Hunink et al have reported a method of correcting for verification bias when some participants receive the gold standard test based on variables other than the diagnostic test result, for example, if patients were sent to biopsy as a result of clinical findings. This method is similar to the method proposed by Begg and Greenes, but includes an additional modeling step [12].

One characteristic of many studies subject to verification bias, particularly those based on screening studies, is that only a very small number of participants with normal diagnostic test results will subsequently receive the gold standard assessment, that is, the number of false negatives is very low. It has previously been demonstrated that sensitivity cannot be accurately estimated in this scenario, even after correction for verification bias[13]. Here, we extend this argument to the area-under-the-receiver-operating-characteristic-curve. We also describe several previously published studies that involved verification bias correction, and examine whether their results might have been influenced by low false negative counts.

## Methods

**Figure 1:**
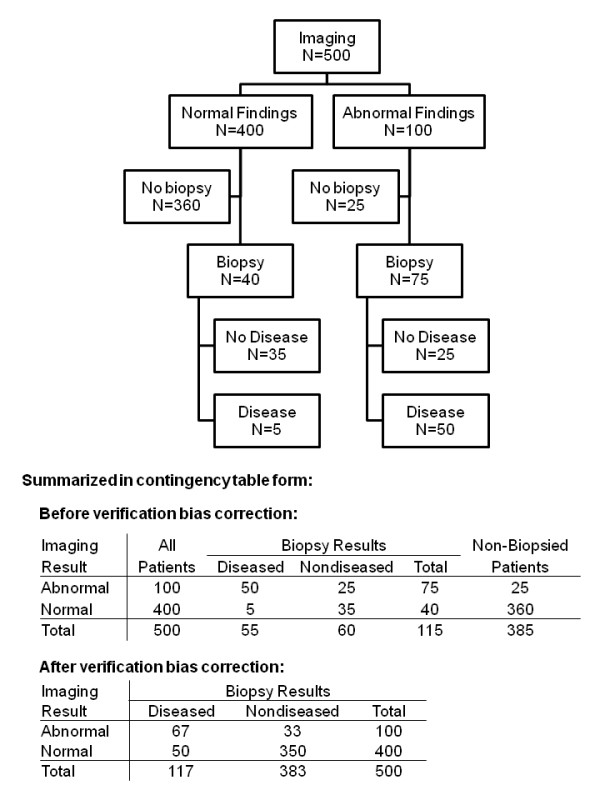
Example of data subject to verification bias.

Take the case of study of screening for cancer, where the aim is to determine the relationship between results of the screening test and true disease status. Patients are screened using an imaging technology (the diagnostic test), and those with abnormal findings recommended for biopsy (the gold standard assessment). A hypothetical example from such a screening program is shown in Figure 1. A total of 500 patients are screened and 100 have abnormal findings. Since those with abnormal findings are strongly recommended to undergo biopsy, 75/100 decide to have a biopsy and 50/75 are confirmed to have disease present. Of the 400 patients with normal findings, 40 are nonetheless biopsied, and 5 are found to have disease.

To estimate the sensitivity and specificity of imaging for detecting cancer, the naive approach would be to use only data from biopsied patients. This results in a sensitivity of 91% (50/55) and a specificity of 58% (35/60). However, it is obvious that patients with unfavorable characteristics (those likely to be both diagnostic and gold standard positive) are overrepresented and patients with favorable characteristics (those likely to be both diagnostic and gold standard negative) are underrepresented in the sample of biopsied patients. As a result, sensitivity is overestimated and specificity underestimated. This is a classic example of verification bias: imaging results are available for all 500 patients, but the gold standard available only for a subset, which is associated with the imaging result. Methods for verification bias correction, following Begg and Greenes [1] are given in the Appendix (see Additional file 1). Using these methods on our example data set, gives a corrected sensitivity of 57% (67/117) and specificity is 91% (350/383). Without verification bias correction, we would have concluded that imaging was highly sensitive but moderately specific when in fact the reverse is true.

Correction for verification bias becomes problematic if small cell counts are encountered. Table 1 gives some different scenarios for the biopsy results of patients with a normal imaging result in our cancer screening example. In the first row, the scenario shown in figure 1, 40 participants with a normal imaging result were biopsied, of which 5 were found with disease – that is, there were 5 false negatives – giving a corrected sensitivity of 57%. In subsequent rows in table 1, we vary the number of false negatives and find that small changes in the data lead to large differences in our estimates: a change from 2 to 1 false negatives, for example, increases sensitivity from 77% to 87%. Clearly no robust statistical method should give such a different result given a change in status for a single patient in a 500 patient study.

**Table 1:** Examples of data subject to verification bias and with a low number of false negatives

Example	Number with normal imaging result and biopsied			Corrected Sensitivity* (%)
		
	Diseased (False negatives)	Nondiseased (True negatives)	Total	
1	5	35	40	57
2	4	36	40	63
3	3	37	40	69
4	2	38	40	77
5	1	39	40	87
6	0	40	40	100

* Corrected for verification bias

A mathematical explanation for this observation is as follows. Consider the formula for the corrected sensitivity given in the appendix (see Additional file 1):

(1) $$\text{Corrected sensitivity}=\frac{{\text{v}}_{11}÷[({\text{v}}_{11}+{\text{v}}_{12})÷{\text{n}}_{1}]}{{\text{v}}_{11}÷[({\text{v}}_{11}+{\text{v}}_{12})÷{\text{n}}_{1}]+{\text{v}}_{21}÷[({\text{v}}_{21}+{\text{v}}_{22})÷{\text{n}}_{2}]}$$

Here, v indicates patients with verified outcome (e.g. a biopsy result); n indicates all patients; the first and second subscripts refer to the test and gold standard results (e.g. imaging and biopsy) respectively; the subscript indicator 1 and 2 refer to test positive/disease and test negative/no disease. The problematic cell is the false negative cell, since participants are rarely verified if they have a strongly negative diagnostic test result; moreover, when these patients are verified, they are most likely to be disease free. It can be seen from (1) that as the false negative cell count (v_21_) approaches zero, the second term in the denominator of (1) also approaches zero, resulting in a corrected sensitivity that approaches 100%.

The sort of small differences in cell count which, as we have shown, can have a marked effect on estimates, are an inevitable consequence of sampling variability. In our principal example, 5 of the 40 patients with negative imaging results had positive biopsy. The 95% confidence interval for this proportion, 12.5%, is 4% to 27%: accordingly it would not at all be unusual if, were we to repeat this experiment, we were to see only 2 of 40 patients with false negative results. In other words, in the imaging example we could have reported a sensitivity ranging from approximately 40% to 75% due to small chance differences in the number of false negatives, and there would be a very wide confidence interval around these estimates.

To investigate further the effects of low false negative counts on sensitivity, and in turn the AUC, we performed the following experiment:

A) We created a simulated data set with 5000 subjects. Both diagnostic and gold standard test results were known for all 5000 subjects, constituting a fully verified data set. Since the gold standard result was known for all subjects, we were able to fix the true AUC to 0.750. Data were simulated according to the specified probability models:

a. The gold standard test result follows a Bernoulli distribution with the mean equal to the incidence of disease, which was set to 10%.

b. The diagnostic test result follows a log normal distribution where the log(test result) has a standard deviation of 1 and a mean of 0 and 1, respectively, for patients with negative and positive gold standard test results.

B) We introduced verification bias to the data in step A such that a certain proportion *v *of participants were verified, where *v *was varied as an experimental parameter. The probability *p *of verification for each subject increased with the diagnostic test result using the formula log [*p*/(1-*p*)] = α + 0.5*d*, where *d *was the decile of the diagnostic test result and the constant α adjusted to fix the overall probability of verification to *v*. This gives the probabilities shown in the top half of table 2. We then applied a correction for verification bias, as shown in the appendix. Note that verification status depended solely on the diagnostic test result, therefore fulfilling the missing at random assumption required for this method. Since the diagnostic test in our simulation has a continuous distribution, the sensitivity and specificity was derived for multiple thresholds by dichotomizing the subjects into abnormal (above the threshold) and normal (below the threshold). A receiver operating characteristics curve was then constructed from these estimates [2] to calculate an AUC corrected for verification bias.

**Table 2:** Probability of verification used in the simulations for each decile of the diagnostic test result

Decile of diagnostic test result	Probability of having the gold standard result (%)		
	
	10% verified	30% verified	60% verified
**Probabilities of verification likely to be encountered in a screening study**			

1	0.6%	3.0%	16.0%
2	0.9%	4.9%	23.9%
3	1.5%	7.8%	34.2%
4	2.5%	12.2%	46.1%
5	4.1%	18.7%	58.5%
6	6.5%	27.5%	69.9%
7	10.3%	38.5%	79.3%
8	15.9%	50.7%	86.3%
9	23.8%	62.9%	91.2%
10	34.0%	73.7%	94.5%

**Probabilities of verification were adjusted such that false negatives were more (less) likely to be present for 10% (60%) verified**			

1	6.4%	--	0.0%
2	7.0%	--	0.0%
3	7.7%	--	0.1%
4	8.5%	--	7.6%
5	9.3%	--	92.4%
6	10.2%	--	99.9%
7	11.1%	--	100.0%
8	12.1%	--	100.0%
9	13.2%	--	100.0%
10	14.4%	--	100.0%

C) We repeated step B five times. Since we introduced verification bias in the same manner each time, we would expect no important differences in data structure between replications. Using the same argument, we would expect no important difference in verification bias corrected AUC unless standard methods were not appropriate for these data.

D) We compared the true AUC from step A to the verification bias corrected AUC corresponding to each replication from step C.

The AUC was calculated using the trapezoid rule, where sensitivity and specificity were estimated (a) over 10 categories based on the deciles of the diagnostic test result and (b) for each unique value of the diagnostic test result using semiparametric efficient estimators – subsequently referred to as the Alonzo-Pepe method -, the latter of which has been shown to have minimal bias when the verification mechanism is known[10]. We specified that the simulated set have 5000 participants with 10% verification since these are common characteristics of large screening studies [4,7,9].

We performed a simulation experiment where we repeated steps A and B 2000 times and report the mean of the true and verification bias corrected AUC, as well as the 2.5^th ^– 97.5^th ^percentiles and coverage. Coverage was the proportion of 95% confidence intervals, constructed using bootstrap methods with 2000 replications, containing the true value of 0.750. We performed this simulation experiment varying the proportion verified (*v *= 10, 30, and 60%). Our intent in varying the proportion verified was to vary the frequency of the cell counts while keeping the relationship between the diagnostic test and outcome the same. For example, with all else being equal, one would be less likely to encounter small cell counts with 60% verified compared to 10% verified. To test whether small cell counts or overall verification rates drove our findings, we repeated our simulations using probabilities of verification as shown in the bottom half of table 2: in this case, the probability of small numbers of false negatives is higher in the scenario with a higher overall verification rate. For the simulations, the AUC was calculated by estimating sensitivity and specificity over 10 categories based on the deciles of the diagnostic test result; we did not calculate the AUC using the Alonzo-Pepe method as it gave similar results. All statistical analyses were conducted using Stata 9.2 (StataCorp, College Station, TX).

## Results

### Simulation studies

Data simulated under the setting of a screening study with 5000 participants are shown in Table 3. Defining "false negatives" as participants below the median diagnostic test level with a positive gold standard result, 99/2500 (4.0%) were false negatives. Table 3 also shows five example runs of our simulation. After verification bias was randomly introduced the first time, only 50 (2% of 2500) participants below the median diagnostic test level had gold standard assessment of which 2 were false negatives. We introduced verification bias to the same simulated data set 4 more times. Comparing the replications, we observe no important differences in the proportion of participants below the median diagnostic test level who subsequently underwent gold standard assessment (1.6% – 2.6%). We also observe that the proportions of false negatives in each replication are consistent with chance when compared to the 4.0% of false negatives in the fully verified data set. Notably, the number of false negatives encountered ranged from 0–4. AUC corrected for verification bias ranged from 0.550 to 0.852. The variation in these results is very large: few estimates of AUC in the medical literature are less than 0.55 or greater than 0.85. In other words, two replications of a screening study could produce results at opposite ends of the extremes of test characteristics. The estimates of AUC using the Alonzo-Pepe method showed similar gross variability, indicating that the variation in results is not explained by categorizing the continuous diagnostic test result in 10 groups. Similar results were observed when varying the true value of the AUC, although less variation was present among highly predictive tests (for example, AUC of 0.9, table 4).

**Table 3:** Example of data generated under the setting of a screening study with 5000 participants and the underlying incidence of disease being 10%

	(1)	(2)			
Data Set	Number with negative diagnostic test and verified	Number of false negatives	Proportion of false negatives (2)/(1)	AUC	
				Categorize in 10 bins	Alonzo-Pepe
Fully verified	2500	99	4.0%	0.750	0.750
With verification bias					
Replication 1	50 (2.0%)	2	4.0%	0.690	0.712
Replication 2	64 (2.6%)	4	6.3%	0.852	0.857
Replication 3	40 (1.6%)	2	5.0%	0.550	0.546
Replication 4	61 (2.4%)	0	0.0%	0.812	0.826
Replication 5	65 (2.6%)	3	4.6%	0.790	0.803

In the fully verified data set, definitive test results were known for all 5000 participants. In the replications with verification bias, only 500 (10%) participants underwent definitive testing. False negatives are defined as verified participants with a diagnostic test result less than the median of the diagnostic test results and with a positive gold standard result. The AUC of the fully verified data set was 0.750; all other estimates are with correction for verification bias.

**Table 4:** Examples of estimates of verification bias corrected AUC when varying the true value of the AUC

	AUC			
	
Fully verified (True value)	0.600	0.700	0.800	0.900
With verification bias correction				
Replication 1	0.284	0.503	0.688	0.873
Replication 2	0.754	0.838	0.742	0.894
Replication 3	0.567	0.578	0.740	0.873
Replication 4	0.689	0.779	0.883	0.920
Replication 5	0.648	0.755	0.856	0.827

The results of the simulation study are shown in table 5. The number of false negatives is shown for a cut-off at the 2^nd ^decile. With 10% of participants verified, 0 false negatives were encountered in 83% of the replications; 1 false negative in 16%, and > 1 false negative in 2%. The proportion of replications with > 1 false negative increased as the percentage verified increased. With 30% of participants verified, 23% of replications had > 1 false negative; this proportion rose to 92% with 60% of participants verified.

**Table 5:** Simulation study with 2000 replications

Percentage Verified	Proportion with *n *false negatives				AUC over 2000 replications		Coverage Probability over 2000 replications
						
	*n *= 0	*n *= 1	*n *= 2	*n *> 2	Mean	2.5^th ^– 97.5^th ^percentile	
**Probabilities of verification likely to be encountered in a screening study**							

True	n/a	n/a	n/a	n/a	0.750	0.728, 0.774	95%
10%	83%	16%	2%	0%	0.758	0.577, 0.860	77%
30%	41%	37%	17%	6%	0.752	0.677, 0.813	89%
60%	1%	6%	12%	80%	0.750	0.713, 0.786	93%

**Probabilities of verification were adjusted such that false negatives were more (less) likely to be present for 10% (60%) verified**							

10%	25%	35%	23%	16%	0.751	0.677, 0.820	93%
60%	98%	3%	0%	0%	0.728	0.552, 0.819	67%

5000 participants are enrolled in a screening study with an underlying incidence of disease of 10%. False negatives are defined as verified participants with a diagnostic test result less than the 20^th ^percentile of the diagnostic test results and with a positive gold standard result.

The verification bias corrected AUC had little bias: the mean over 2000 replications was generally close to the true AUC of 0.750. As expected, presence of verification bias increased the amount of variability associated with the AUC. With full verification, the 2.5^th ^– 95^th ^percentiles of the AUC over 2000 replications were 0.728 – 0.774; with 10% of participants verified, which is commonly observed in screening settings, this increased to 0.577 – 0.860. The width of this interval, 0.28, covers approximately 60% of all possible values of AUC. One way of illustrating these results is to note that a study with only 100 patients and a 10% event rate has a confidence interval for AUC of approximately 0.3: thus a study with 5000 patients that is subject to verification bias has equivalent statistical precision to one 98% smaller. Coverage was only 77% when 10% of patients were verified. As expected, both variability and coverage improved with a higher proportion verified: with 60% of participants verified, the 2.5^th ^– 95^th ^percentiles of the AUC were 0.713 – 0.786 and coverage was 93%.

Table 5 gives the results for the scenarios in the bottom half of table 2, where the number of false negatives is higher when the overall verification rate is lower. It is clear that the false negative count determines the value of verification bias correction: even if, overall, a reasonable number of patients are verified (60%), our estimates have poor properties when there are few false negatives.

### Published example 1 – HPV, cervical cancer example

In 2004, Dannecker et al examined the sensitivity of human papillomavirus (HPV) DNA on self-collected vaginal swabs for the diagnosis of cervical cancer [8]. The study included 435 participants in total, of whom 122 (28%) underwent colposcopy, the gold standard assessment. The paper reported a sensitivity of 100% after verification bias correction, yet it is clear from examination of the paper that there were no false negatives. If instead there was 1 false negative (e.g. 1 participant with a negative diagnostic test result who was positive on colposcopy), the sensitivity after verification bias correction would be markedly reduced, from 100% to 70%. Since there were zero false negatives, verification bias corrected confidence intervals cannot be derived (the authors did not provide confidence intervals, and did not mention the variability associated with the estimated sensitivity).

### Published example 2 – CAD, Single Photon Emission Computed Tomography

In 2002, Miller et al examined the sensitivity of single photon emission computed tomography (SPECT) for the diagnosis of coronary artery disease (CAD) [7]. The study included 14,273 participants in total, of whom 1853 (13%) underwent coronary angiography, the gold standard assessment. This paper reported a sensitivity of 65% (95% confidence interval 63 to 68%) after verification bias correction. There were 32 false negatives in the study. Small changes to the number of false negatives did not have a substantive difference in this example. For example, we estimate that changing the number of false negatives to 25 would result in a corrected sensitivity of 68%, and to 40 would result in a corrected sensitivity of 62%; these estimates are not importantly different from the reported sensitivity of 65%, and are nearly within the reported 95% confidence interval. From reviewing the statistical methods of the manuscript, we hypothesize that the reported confidence intervals are too narrow because the analysis included stepwise model selection, which was not replicated during the bootstrap procedure.

### Published example 3 – PSA, prostate cancer example

In 2003, Punglia et al reported the AUC of a PSA test for prostate cancer diagnosis in a screening study conducted in the United States [4]. Of 6,691 men with PSA results, 705 (11%) underwent biopsy of the prostate. The investigators performed a verification bias correction and reported the AUC of the PSA test for men aged < 60 years to be 0.86. From the presented tables and figures for men aged < 60 years, we observe that the corrected sensitivity remained at 100% for PSA < 0.9 ng/ml. This implies that there were no false negatives among men aged < 60 years with PSA < 0.9 ng/ml. Using simple assumptions (the median PSA among all 4556 men < 60 screened being 0.9 ng/ml and holding the specificity at this PSA level around 55%, as reported), and making a small change in false negatives, from 0 to 1, the corrected sensitivity drops to 80%. Had the AUC been calculated using 80% sensitivity instead of 100% sensitivity for PSA < 0.9 ng/ml, then the corrected AUC would be 0.78 instead of 0.86. Confidence intervals were not provided by the author for any sensitivity or AUC estimates.

## Discussion

Verification bias is present in studies where only a subset of subjects receives the gold standard confirmation of disease status and where the likelihood of the gold standard confirmation depends on the diagnostic test result. These two conditions are often met in screening studies. When verification bias is not accounted for, reported sensitivity is inflated and specificity is understated. It is possible to obtain verification bias-corrected estimates of sensitivity and specificity if at least 1 subject with a negative screening test receives the definitive testing.

Several systematic reviews have investigated the prevalence of verification bias in diagnostic studies. In a review of pediatric studies published from 1987 to 1989, 40% (15/42) were found to be verification bias[14]. Reviewing all diagnostic test studies published from 1978 to 1993, correction for verification bias was performed in 46% (51/112) studies. Notably, the proportion of studies that corrected for verification bias significantly increased over time: 29% from 1978–1981 and 62% from 1990–1993. Finally, in a review of studies examining diagnostic tests for cancer published from 1990 to 2003, 40% (10/25) at least mentioned verification bias as a potential source for bias[15].

It is important to recognize not only the need for but also the limitations of verification bias correction. In this paper, we illustrate that standard methods for verification bias correction are not adequate when there are few false negatives. This situation is commonly encountered in screening studies [4,8]. In these cases, verification bias correction would have led to dramatically different results given very small changes in the number of false negatives. Most skilled statisticians would be wary about applying statistical methods when there are low cell counts, and would therefore be concerned about the adequacy of verification bias correction in this circumstance. Yet we have shown that verification bias correction is often applied regardless of low numbers of false negatives. In the HPV example, the authors concluded that the HPV DNA test had excellent sensitivity, when in fact the reported sensitivity of 100% would have been 70% were a single patient to be reclassified. In the PSA screening example, the AUC could have been 0.78 instead of 0.86 if a single cancer were found among the estimated 2300 men with PSA < 0.9 ng/ml. Although PSA is a screening test with a continuous distribution, it should be noted that verification bias-corrected estimates should not be obtained for PSA levels below which no man receives a biopsy. Indeed, using data from the Prostate Cancer Prevention Trial[16], a study in which men were biopsied irrespective of PSA level and thus not subject to verification bias, we might estimate well over 100 false negatives in the PSA < 0.9 ng/ml group.

One possible solution to low false negative rates would be to provide confidence intervals, so that the width of the confidence interval can give insight into the certainty of the point estimate. Begg and Greenes provide formulas to calculate confidence intervals in the presence of verification bias, however, these formulas are based on asymptotic theory. In the case of few false negatives, the resulting confidence intervals will be insufficiently wide due to the uncertainty of the negative predictive value[13]. An obvious distribution-free method to calculate confidence intervals is by bootstrapping. However, since the false negative rate is underestimated in data subject to verification bias, we cannot be certain that resampling methods will sample from the true distribution of false negatives, in particular when the diagnostic test has a continuous distribution. For example, consider the case where the diagnostic test is continuous and there is only one false negative with a test result t = t* below a threshold value of the test *q*. Resampling methods will give a value t = t* for all diseased subjects below *q*, which is not the true distribution. As a result, bootstrap methods will provide overly narrow confidence intervals (table 5). In the HPV example, since the actual data did not contain any false negatives, no bootstrap sample would contain any false negatives; hence, the sensitivity would be overestimated in all bootstrap samples. Therefore, we do not believe that bootstrap methods to produce verification bias corrected confidence intervals are appropriate.

We have described the application of certain statistical methods in the presence of verification bias, and the problems with these methods when the analysis data set contains few false negatives. In other words, this manuscript focuses on the analytic phase of the study, and not the design phase. If it is known in advance that verification bias will be an issue, then a possible solution would be to design the study such that a random sample of test-negative subjects to undergo disease verification. The size of this sample would need to be sufficiently large to provide an adequate number of false negatives to estimate sensitivity, which may be difficult to determine in the original study design. In the CAD example, less than 2% of the test negative participants (97/6745) were in the verified sample; a higher percentage of test negatives in the verified sample would be required in a smaller study. Although verification bias corrected estimates of diagnostic accuracy would be improved, this would come at a cost: subjects with no indication of disease would undergo unnecessary procedures, possibly resulting in complications and discomfort, not to mention the monetary cost of the additional procedures. The appropriateness of randomly selecting test-negative subjects to receive the gold standard assessment will be specific to the disease (for example, the incidence of disease and the invasiveness of the gold standard assessment. Since the focus of this manuscript is data analysis, and not study design, we have not made specific recommendations on this point.

For our simulations, we used data sets with 5000 subjects, a 10% incidence of disease and a verification rate as low as 10%. These numbers were chosen to reflect the typical parameters of screening studies. For example, in one cervical cancer study, 364 of 4761 (8%) women underwent biopsy [9]; the biopsy rate was 705 of 6691 (11%) of men in a prostate cancer screening study; in a cardiovascular study 340/3679 (9%) patients underwent angiography for disease status confirmation [5]. With respect to the true incidence of disease, this is naturally difficult to determine. As such, we conservatively chose a high incidence of 10% (note that, for example, the lifetime incidence of cervical cancer is less than 1%[17]). It thus seems plausible that many screening studies would be prey to the problem of low false negative rates. The published examples (3.2 – 3.4 above) provide additional evidence on this point.

Previous mention has been made to the inadequacy of verification bias correction in the presence of low false negatives. In their critique of a novel imputation method, Hanley et al noted that sensitivity was 100% when there were no false negatives, even after verification bias correction. They cautioned against verification bias correction when one or more of the cells in the verified sample is zero [18]. Pepe has previously noted that verification bias corrected estimates of sensitivity are not robust to low numbers of false negatives[13]. We further demonstrate that standard methods to correct for verification bias, which have been widely used with zero false negatives, will produce unreliable estimates of sensitivity and AUC. Ultimately, verification bias is a missing data problem and we hypothesize that no correction method will be able to overcome a cell count of zero.

## Conclusion

The characteristics of diagnostic tests have important clinical implications. From these results, clinicians decide whether or not to use a diagnostic test, and if so, how it should be used, such as what PSA cutoff to use to recommend patients for biopsy. Screening programs are designed such that few false negatives are encountered. When there are few false negatives, standard methods for verification bias correction are inadequate. If these methods are to be used, then at a minimum verification bias corrected confidence intervals should be provided for all estimates of diagnostic accuracy. Investigators should be cautioned when using such methods: there are no "free lunches" in statistics, and we should certainly be skeptical of methods that appear to get something from nothing.

## Competing interests

The authors declare that they have no competing interests.

## Authors' contributions

AMC and AJV conceived of the study, and participated in its design and coordination and helped to draft the manuscript. AMC performed the statistical analyses. All authors read and approved the final manuscript.

## Pre-publication history

The pre-publication history for this paper can be accessed here:



## Supplementary Material

Additional file 1 **Appendix verification bias** Click here for file
