# Supplementary material for: Statistical methods to correct for verification bias in diagnostic studies are inadequate when there are few false negatives: a simulation study
Source: BMC Med Res Methodol. 2008 Nov 11;8:75. doi: 10.1186/1471-2288-8-75 (PMC2600821; doi:10.1186/1471-2288-8-75)
Supplement: Additional file 1 — Appendix verification bias [file 1471-2288-8-75-S1.doc]

**Appendix**

Generalized formulae for verification bias correction per Begg and Greenes [1] [2].

A typical representation of data subject to verification bias is shown below:

| Diagnostic |  | All Patients |  | Verified Patients | | |  | Nonverified Patients |
| --- | --- | --- | --- | --- | --- | --- | --- | --- |
| Test result |  |  |  | Diseased | Nondiseased | Total |  |  |
| Abnormal |  | n_1_ |  | v_11_ | v_12_ | v_11_ + v_12_ |  | n_1_ – (v_11_ + v_12_) |
| Normal |  | n_2_ |  | v_21_ | v_22_ | v_21_ + v_22_ |  | n_2_ – (v_21_ + v_22_) |

The entire cohort is divided into two groups, “verified” and “nonverified”. The empirical probability of verification (i.e. receiving the gold standard test) is calculated for each test result category. Within each test result category, the observed frequency counts among verified patients are divided by the empirical probability to obtain unbiased estimates of the frequency counts encountered had all patients received the gold standard test, as shown below:

| Diagnostic  Test result |  | Empirical probability  of verification |  | Corrected for verification bias  (unbiased estimates) | |
| --- | --- | --- | --- | --- | --- |
|  |  |  |  | Diseased | Nondiseased |
| Abnormal |  | p_1_ = (v_11_ + v_12_) ÷ n_1_ |  | v'_11_ = v_11_ ÷ p_1_ | v'_12_ = v_12_ ÷ p_1_ |
| Normal |  | p_2_ = (v_21_ + v_22_) ÷ n_2_ |  | v'_21_ = v_21_ ÷ p_2_ | v'_22_ = v_22_ ÷ p_2_ |

Sensitivity and specificity are derived from this table [1]. The method of correction when other variables are used to select participants to receive gold standard assessment has one modification: the empirical probability of verification is replaced by the expected probability of verification obtained from a logistic regression model [10].
